# Supplementary material for: A Comparative Study of the Synthesis and Hydrolysis of sym-Triaminobenzene Homologues
Source: Molecules. 2022 Dec 6;27(23):8595. doi: 10.3390/molecules27238595 (PMC9737077; doi:10.3390/molecules27238595)
Supplement: Supplementary file 1 [file molecules-27-08595-s001.zip › molecules-2078910-supplementary.pdf]

Supplementary Materials

A Comparative Study of the Synthesis and Hydrolysis Processes of *sym*-Triaminobenzene Homologues

Irina A. Shchurova*, Natalia A. Alekseyeva, Sergey V. Sysolyatin and Valeriy V. Malykhin

Institute for Problems of Chemical and Energetic Technologies, Siberian Branch of the Russian Academy of Sciences (IPCET SB RAS), Biysk 659322, Russia

Correspondence. E-mail: shchurova_irina@mail.ru

Table of contents

^{1}H and ^{13}C NMR of compound **2b** S2

^{1}H and ^{13}C NMR of compound **2c** S3

^{1}H and ^{13}C NMR of compound **2d** S4

^{1}H and ^{13}C NMR of compound **3b** S5

^{1}H and ^{13}C NMR of compound **3c** S6

^{1}H and ^{13}C NMR of compound **3d** S7

^{1}H and ^{13}C NMR of compound **4** S8

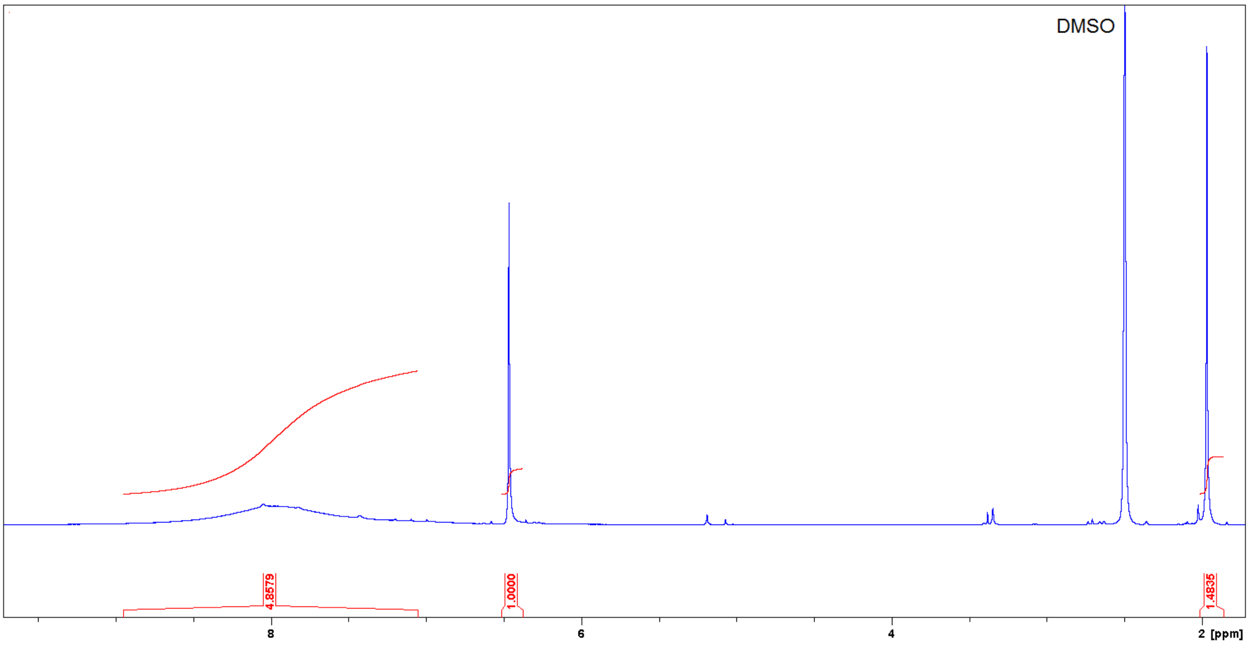

Figure S1. ^{1}H NMR (DMSO-d$_6$) spectrum of compound **2b**

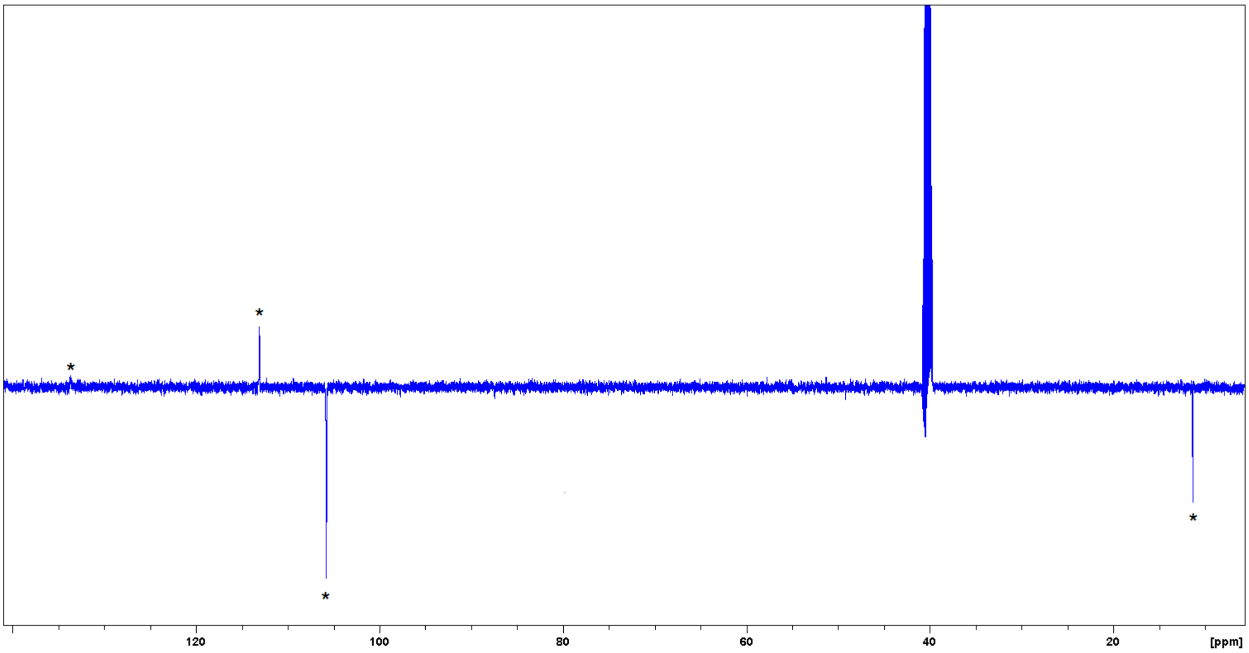

Figure S2. ^{13}C NMR (DMSO-d$_6$) spectrum of compound **2b**

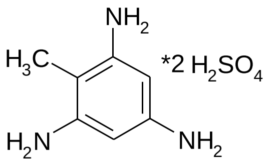

2,4,6-Triaminotoluene disulfate (2b)

^{1}H NMR (500 MHz, DMSO-d$_6$) δ_H ppm: 1.97 (s, 3H, CH_3), 6.47 (s, 2H, CH), 7.95 (bs, NH).

^{13}C NMR (125 MHz, DMSO-d$_6$) δ_C ppm: 11.4, 105.8, 113.1, 133.7.

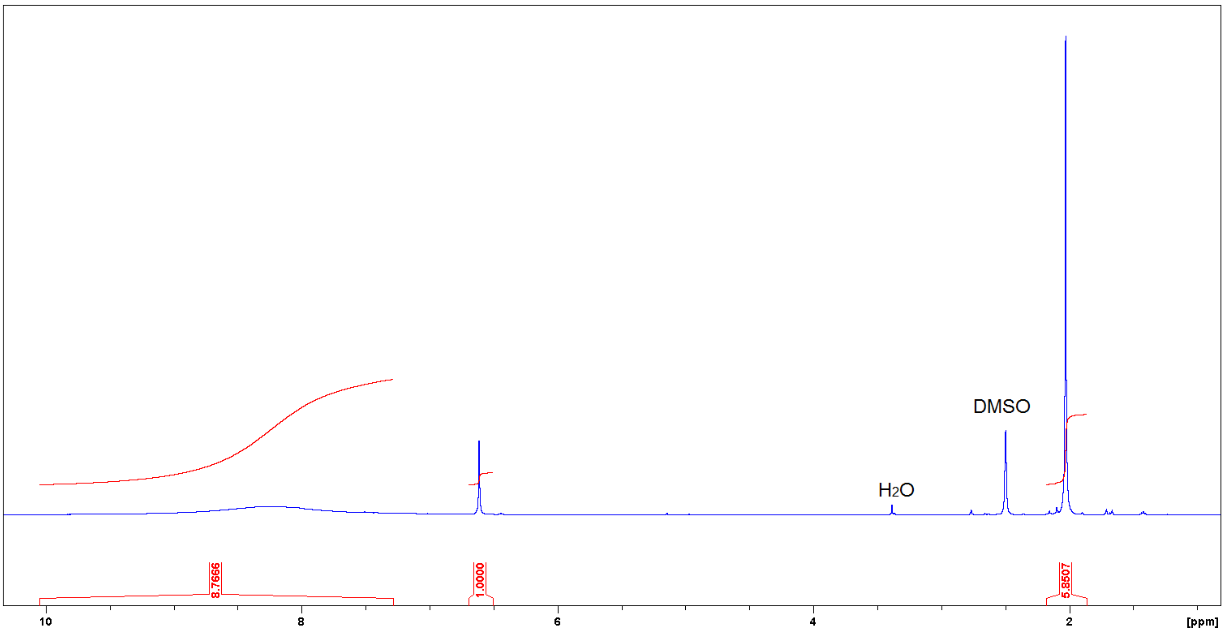

Figure S3. ^{1}H NMR (DMSO-d_6) spectrum of compound **2c**

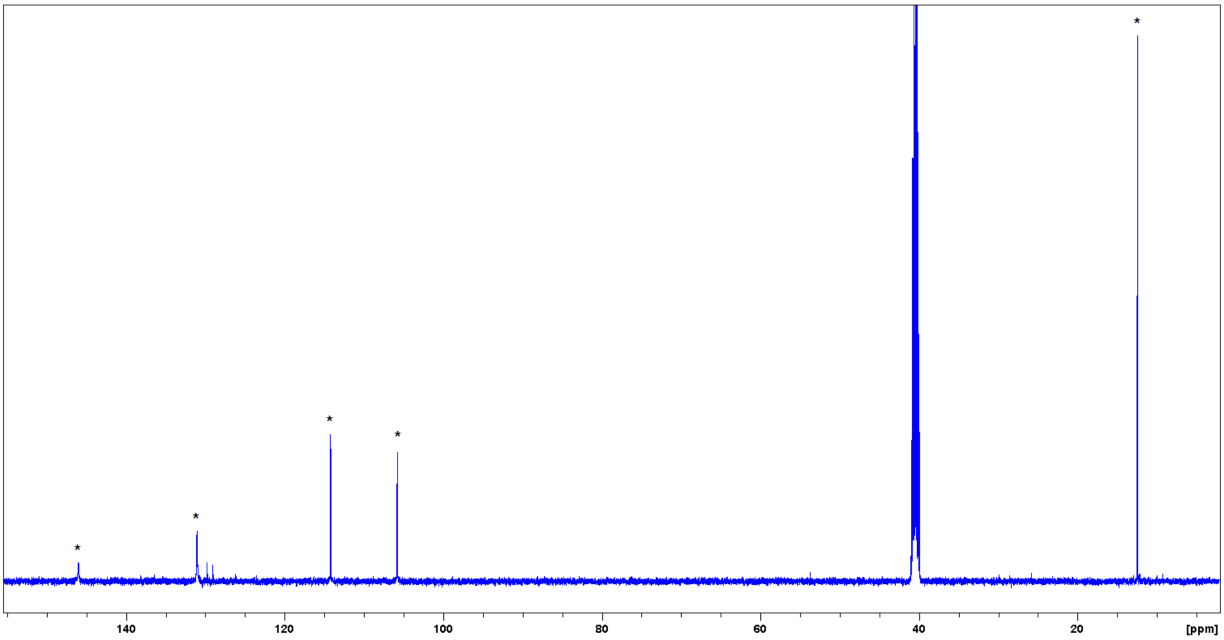

Figure S4. ^{13}C NMR (DMSO-d_6) spectrum of compound **2c**

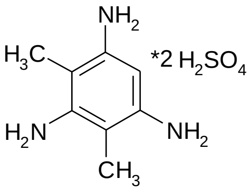

2,4,6-Triaminoxylene disulfate (2c)

^{1}H NMR (500 MHz, DMSO-d_6) δ_H ppm: 2.03 (s, 6H, CH_3), 6.61 (s, 1H, CH), 8.25 (bs, NH).

^{13}C NMR (125 MHz, DMSO-d_6) δ_C ppm: 12.5, 105.8, 114.2, 131.1, 146.1.

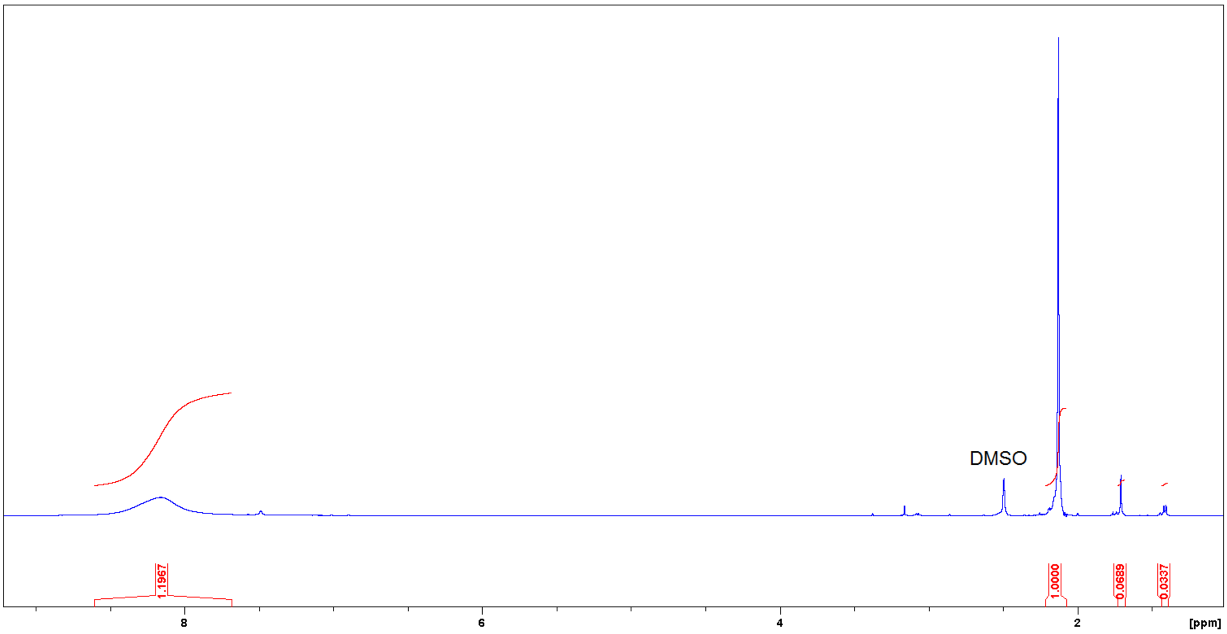

Figure S5. ^{1}H NMR (DMSO-d_6) spectrum of compound **2d**

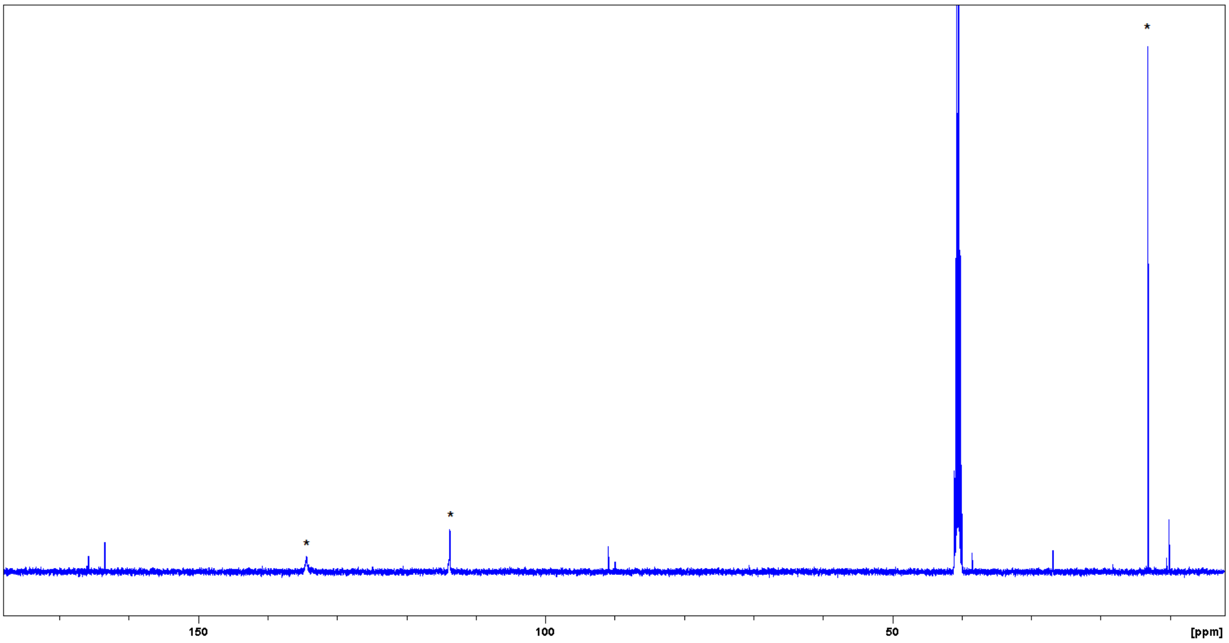

Figure S6. ^{13}C NMR (DMSO-d_6) spectrum of compound **2d**

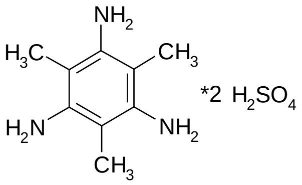

2,4,6-Triaminomesitylene disulfate (2d)

^{1}H NMR (500 MHz, DMSO-d_6) δ_H ppm: 2.13 (s, 9H, CH_3), 8.17 (bs, NH).

^{13}C NMR (125 MHz, DMSO-d_6) δ_C ppm: 13.1, 113.7, 134.4.

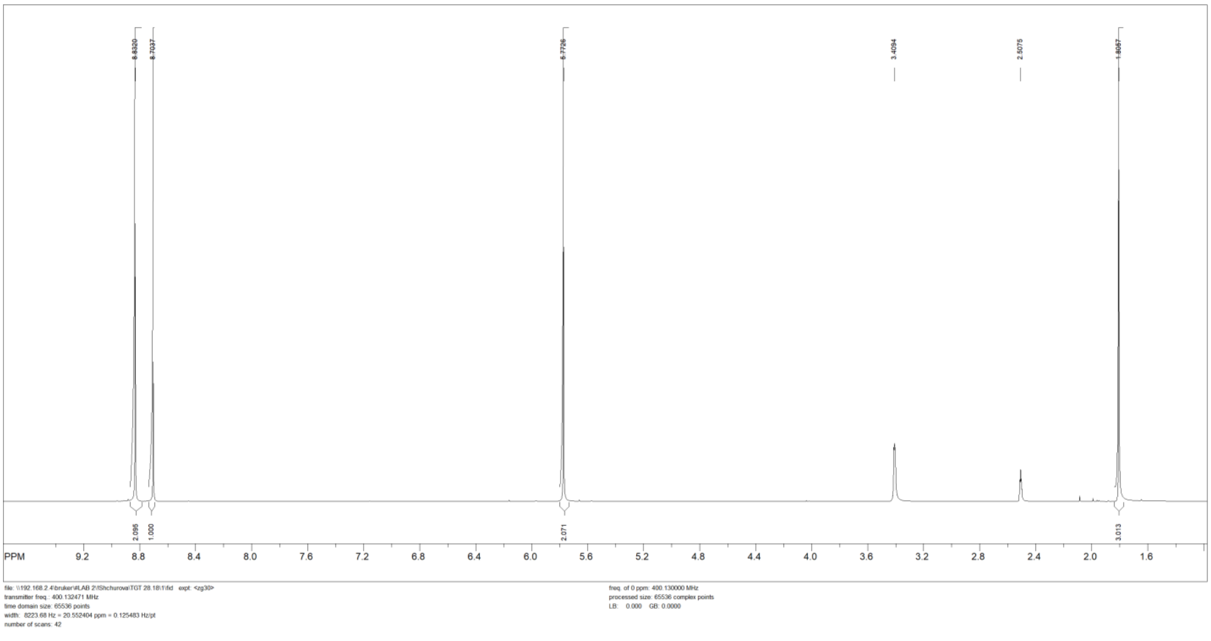

Figure S7. ^{1}H NMR (DMSO-d_6) spectrum of compound **3b**

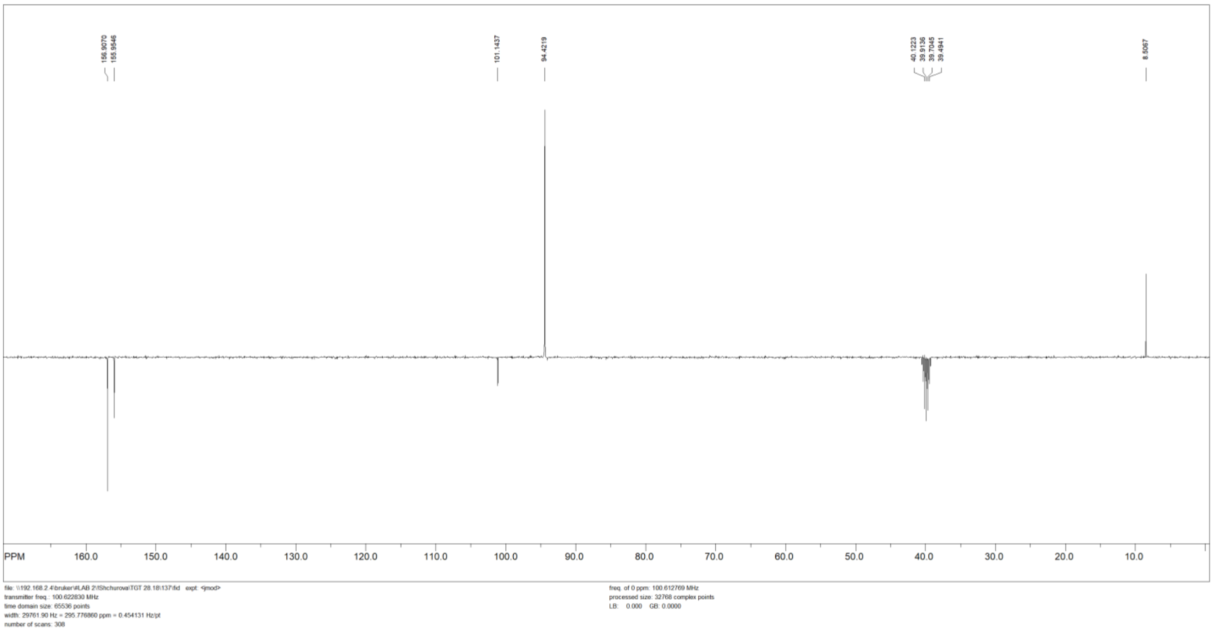

Figure S8. ^{13}C NMR (DMSO-d_6) spectrum of compound **3b**

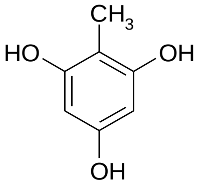

2-Methyl phloroglucinol (3b)

^{1}H NMR (400 MHz, DMSO-d_6) δ_H ppm: 1.81 (s, 3H, CH_3), 5.77 (s, 2H, Ar), 8.70 (s, 1H, OH), 8.83 (s, 2H, OH).

^{13}C NMR (100 MHz, DMSO-d_6) δ_C ppm: 8.5, 94.4, 101.1, 156.0, 156.9.

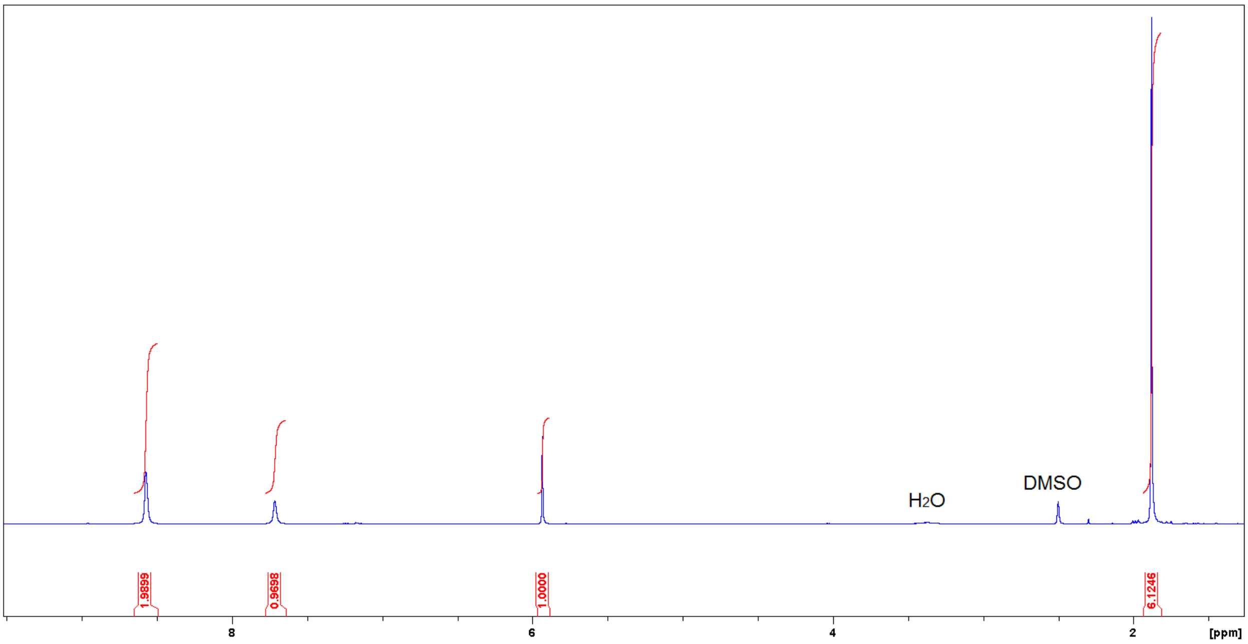

Figure S9. ^{1}H NMR (DMSO-d$_6$) spectrum of compound **3c**

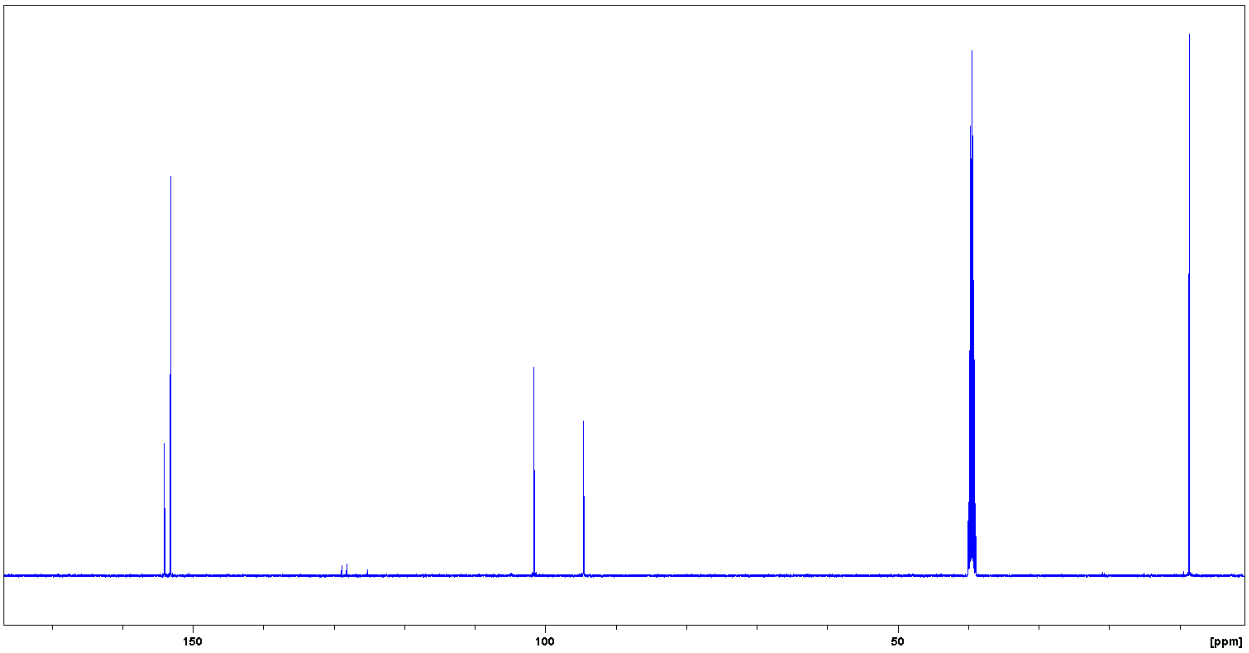

Figure S10. ^{13}C NMR (DMSO-d$_6$) spectrum of compound **3c**

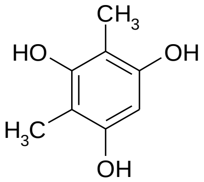

2,4-Dimethyl phloroglucinol (3c)

^{1}H NMR (500 MHz, DMSO-d$_6$) δ_H ppm: 1.88 (s, 6H, CH_3), 5.93 (s, 1H, Ar), 7.72 (s, 1H, OH), 8.58 (s, 2H, OH).
^{13}C NMR (125 MHz, DMSO-d$_6$) δ_C ppm: 8.7, 94.6, 101.6, 153.2, 154.1.

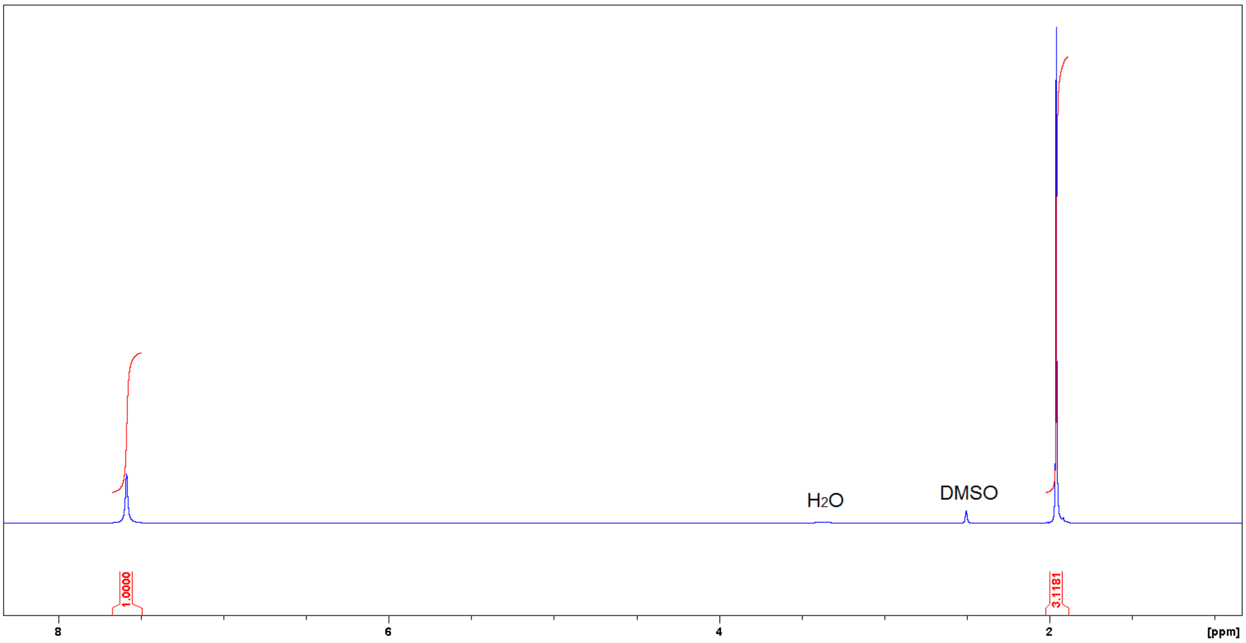

Figure S11. ^{1}H NMR (DMSO-d_6) spectrum of compound **3d**

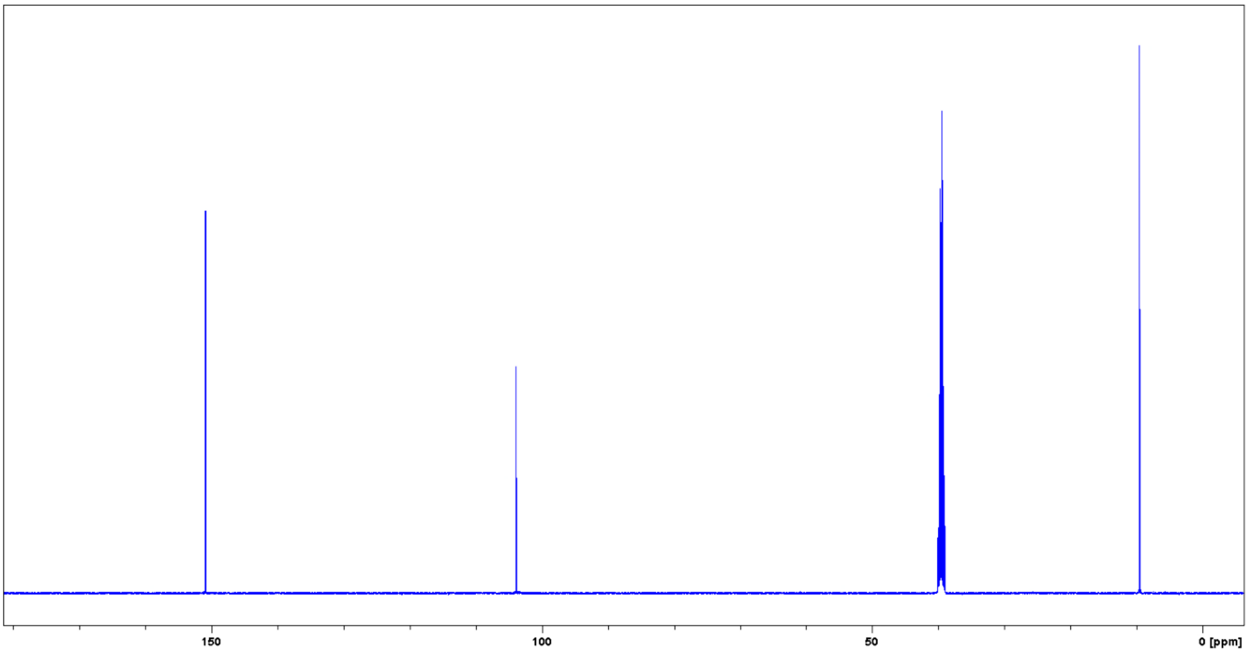

Figure S12. ^{13}C NMR (DMSO-d_6) spectrum of compound **3d**

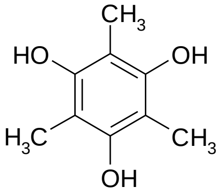

2,4,6-Trimethyl phloroglucinol (3d)

^{1}**H NMR** (500 MHz, DMSO-d_6) δ_H ppm: 1.95 (s, 9H, CH_3), 7.59 (s, 3H, OH).
^{13}**C NMR** (125 MHz, DMSO-d_6) δ_C ppm: 9.5, 103.9, 150.9.

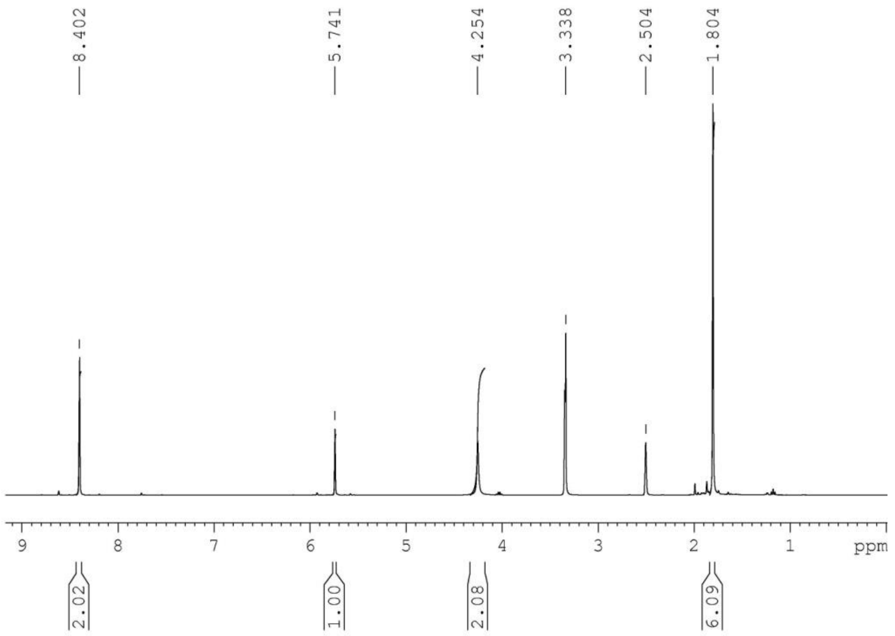

Figure S13. ^{1}H NMR (DMSO-d_6) spectrum of compound **4**

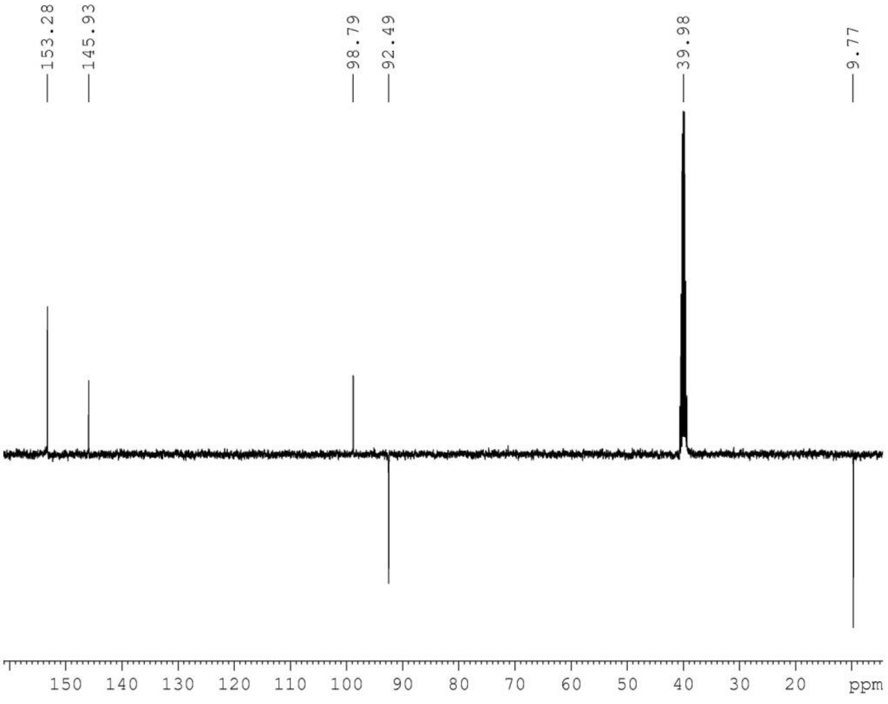

Figure S14. ^{13}C NMR (DMSO-d_6) spectrum of compound **4**

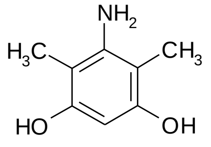

2-Amino-4,6-dimethylphloroglucinol (4)

^{1}**H NMR** (400 MHz, DMSO-d_6): δ_H ppm: 1.80 (s, 6 H, CH_3), 4.25 (s, 2 H, NH_2), 5.74 (s, 1 H, Ar-H), 8.40 (s, 2 H, OH).
^{13}**C NMR** (125 MHz, DMSO-d_6): δ_C ppm: 9.8, 94.5, 98.8, 145.9, 153.3.
